# 

**Published:** 1878-12

**Authors:** 


### INDEX TO VOLUME XXXVII.

PAGE.

A.

Abscess, lumbar, the necessity of more heroic treatment, E. Andrews, M.D. .............. 357
Acne, on proper use of term, Dr. G. H. Fox .................. 370
Adder, spreading, is the bite of, venomous, J. Schneck ....... 585
Address, president American Medical Association, Dr. T. G. Richardson ................ 26
Adhesive splints, Dr. J. D. Skeer's, A. H. Foster, M.D... 71
Advertisements, professional, E. Ingals, M. D ............... 302
Atkinson, W. A., M.D., inflammation ..................... 348
Akystopeurastic, Dr. N. Senn... 396
Alcohol, physiological action of, Dr. Carpenter ........... 217
Allaire, P. A., treatment of bowel affections of infancy and childhood ..................... 262
Alopecia and pityriasis capitis, treatment of, Malassez ....... 102
Anæsthetic, new, M. Paul Bert. 87
Andrews, Edmund, M.D.:
The necessity of more heroic treatment in cases of lumbar abscess ................... 357
Calculus of size of a grain of wheat detected by a new auscultating searcher... ... 260
Two cases of lithotomy...... 259
Aneurism of femoral artery cured by compression, F. E. Waxham, M.D ............. 70
Antiseptic treatment of burns, Bush ...................... 101
Argenti nitras, elastic crayon of. 189
Arnica, treatment of boils by, N. Planat .................. 107
Atkinson, M.D., I. E., the pigmentary syphiloderm........ 340

PAGE.

B.

Belfield, M.D., W. T., migration of leucocytes in passive hyperæmia ...................... 247
Bone, certain points in the pathology of, especially tubercle, Dr. H. H. Smith ............ 34
Board of Health, Michigan State ........................ 604
Boils, treatment of with arnica, M. Planal .................. 107
Bowel affections of infancy and childhood, treatment of ..... 160
Bowel affections of infancy and childhood, treatment of, P. A. Allaire, M.D ................ 262
Bridge, Norman, M.D., unusual distension of the cavity of the cervix uteri, with spasmodic contraction of the internal os, in a case of miscarriage ..... 487
Brown-Séquard, Dr., and his recent lectures, by J. S. Jewell, M.D ................ 1
Bubo, abortive treatment of, M. Malplaquet ................. 210
Burns, treatment of, by sodic bicarbonate ................ 647
Burns, antiseptic treatment of, Bush ..................... 101
Byford, H. T., M.D., a case of partial inversion of uterus... 388
Byford, W. H., M.D.:
Dermoid tumors of uterus... 596
Rapid dilatation of the female urethra in the diagnosis and treatment of chronic cystitis. 10

C.

Cancer of Pylorus, codeia in... 657
Cæsarean section, a successful case, Prof. Carl Braun ....... 293
Cæsarean section, by Prof. Breisky, of Prague ............... 608

PAGE.

Caldwell, W. S., M.D.:
The use of the hydrate of chloral per rectum........ 515
Correspondence............. 515
The management of fevers in Germany................. 173
Correspondence, medical men and matters in Vienna. ... 289
Calculi cystine, Dr. Southam... 330
Calculi vesical, spontaneous disintegration of, Dr. Ord.... 330
Calculus, renal, largely composed of indigo............. 650
Calculus of the size of a grain of wheat detected by the new auscultating searcher, Edmund Andrews, M.D............... 260
Caldwell, W. S, M.D., clinical notes from Prague and Berlin, Vienna,........173, 289, 515, 608
Carbolic acid poisoning, antidote for, Prof. Bauman .... 557
Carcinoma, action of parenchymatous injections of glacial acetic acid, Dr. Gies......... 647
Catarrh, chronic, of stomach, Dr. W. B. Neftel............ 206
Castration, application of ligatures after................ 212
Cervical canal, instruments for the rapid and forcible dilatation of, by H.T. Hanks, M.D. 658
Cervix uteri, Emmet's operation on, by Prof. Breisky, of Prague..................... 609
Children, nursing of, Dr. Brochin............................ 201
Chloral Hydrate, use of per rectum. W. S. Caldwell, M.D.... 515
Chloral hydrate, locally for warts....................... 657
Cholagogues, action of, Prof. Rutherford.................. 556
Cholecystotomy, Dr. Sims...... 329
Chorea minor, propylamine in Dr. Pürckhauer............ 557
Chorion, cystic degeneration of, H. L. Harrington, M.D....... 387
Circulation, influence of position on, Mr. Lister ............. 430
Code of ethics, H. M. Lyman, M. D...................... 502
Cold and heat, action of on organism, P. Delmas.......... 199
Collodion in seasickness, Dr. Laederich................ 325
Compression of the common iliac, Dr. Davy ............. 208
Conia, therapeutic employment of M. Tyryakian............ 553

PAGE.

Convulsions, Puerperal attended with rupture of uterus and recovery, A. C. Rankin, M.D.. 581
Consumption in the northwestern states, H. A. Johnson, M.D. 133
Consumption, a conspectus of the different forms of, Roswell Park, M.D.................. 228
Corpuscles, concerning white, Burden-Sanderson........... 429
Cornea, hernia of, Dr. F. C. Hotz 564
Cornea and conjunctiva, incrustations of lead upon, M. Galezowski.................... 444
Cranium, experimental investigations on the variations of the volume of, M LeBon.... 319
Cruse, T. K., M.D., medical education in France........... 273
Cutter, E., M.D., lecture on morphology of blood in syphilis. 67
Cystitis chronic, rapid dilatation of the female urethra, in diagnosis and treatment of, by Wm. H. Byford, M. D........ 10

D.

Day, W. H., M.D., the sequel and fatal termination of case of lymphatic enlargement right leg and thigh............... 572
Diabetes, its connection with pruritus vulvæ, Dr. Wilshire. 207
Diarrhœa, oxide of zinc in..... 555
Diphtheria, therapeutics of, durin year 1877, Dr. Ferraud.... 652
Diphtheritic paralysis, lesions of the anterior roots in, Déjerine ........................ 95
Dysmenorrhœa, pathology of membranous, Dr. Cory........ 381

E.

Eclampsia, relieved by intravenous injection of hydrate of chloral, M. Bellmunt..... 214
Editorial:
Valedictory of the year ..... 616
Secular press on the treatment of yellow fever........... 404
The late agitation arising from an advertisement ......... 401
Two pictures................ 531
Elephantiasis, nerve section for, Dr. Morton ................ 209
Emphysema artificial, as an aid to operation, Dr. Roucière.. 208
Epithelium and its performances, Dr. Heitzmann....... 371

PAGE.

Erysipelas:
Treatment of in hospital at Prague and Berlin......... 610
Treatment of, by hypodermic injections of carbolic acid, H. Hueter................ 443
Eserine and pilocarpine, action of, in hernia of the cornea, Dr. F. C. Hotz.............. 564
Etheridge J. H., M.D., medical gynæcology.............. 464
Ether v. chloroform .......... 393
Exophthalmic goitre cured by galvanization of the sympathetic, Dr. Ancona.......... 98

F.

Femur fractures of, treatment of in Berlin hospitals....... 613
Fermentation, paper on, Dr. H. M. Lyman................. 68
Fevers, management of in Germany, W. S. Caldwell, M.D.. 173
Fibroid of uterus, Hegar's operation for, Dr. N. Senn.. 395
Flood, J. Ramsey, M.D., correspondence ................. 399
Foster, A. H., M.D., Dr. J. D. Skeer's adhesive splints..... 71
Fractures un-united, treatment of, Mr. Fitzgerald.......... 514
Fractures of the femur, treatment of in Berlin hospitals. 613
Fracture, a case of un-united, Dr. Max Schuller........... 96
Fungoid neoplasm, a case of inflammatory, Dr. L. A. Duhring...................... 377

G.

Gunn, Moses, M.D., correspondence...................... 397
Gynæcology, medical, J. H. Etheridge, A.M., M. D...... 464
Gangrene, a case of symmetrical g. of the fingers, I. F. Stewart M.A., and Noble Holton, M.D ..................... 587
Gangrænopsis, case of, Dr. H. S. Piffard.................. 380
Gastric juice, physiology of, M. Grichet.................... 84
Gillespie, S. W., salicylic acid in acute rheumatism........ 148
Glaucoma and increased ocular tension .................... 445
Goitre exophthalmic cured by galvanization of the sympathetic, Dr. Ancona.......... 98

PAGE.

Gradle, H., M.D.:
On some novel modes of relief in certain visual defects... 363
The functions of the semicircular canals............ 120
Gravel, removal of from bladder, M. Dubuc ............. 441

H.

Hæmorrhage, post partum, a new device for arresting, Dr. Christie .................... 647
Hanks, H. T., M.D., description of instruments for rapid and forcible dilatation of cervical canal ...... ............... 658
Harrington, H. L., M.D.:
H podermic use of morphia. 73
Cystic degeneration of chorion 387
Huse, E. C., M.D., a new mode of arresting nasal and uterine hæmorrhage................ 170
Heat and cold, action of on the organism, P. Delmas........ 199
Hæmorrhage, a new mode of arresting nasal and uterine, E. C. Huse, M.D.............. 170
Hernia strangulated, ergotine in...................... 212
Hernia of cornea, Dr. F. C. Hotz...................... 564
Heterodon, or spreading adder, is the bite of venomous? I. Schnéck, M.D............. 585
Hiccough, obstinate, treatment of, M. Favier.............. 207
Hip joint, exsection of, Dr. L. A. Sayre................. 435
Hirsuties, treatment of, Dr. W. A. Hardaway............... 379
Holton, Noble, M.D., and J. T. Stewart, M.D., a case of symmetrical gangrene of fingers. 587
Holmes, E. L., M.D., ophthalmology as related to general medicine................... 143
Homœopathy, the critical period of, by H. M. Paine, M.D..... 636
Hospital, Mercy, reports of cases ......................... 70, 259
Hotz, F. C., M.D.:
Chronic otorrhœa; caries of petrous bone; fatal meningitis ..................... 392
Hernia of cornea............ 564
Translation from German of Dr. Liel, under what conditions can the nasal douche be employed without danger?. 280

PAGE.

Hyde, J. N., M.D., the nursemaid and the mother of the syphilitic child............. 452
Hygiene, address on, by Dr. J. L. Cabell.................... 53
Hyperæmia, passive, migration of leucocytes in, W. T. Belfield, M.D.................. 247
Hypodermic use of morphia, H. L. Harrington, M.D. ........ 73

I.

Inflammation, W. A. Atkinson, M.D........................ 348
Ingals, E., M.D., professional advertisements.............. 302
Intestinal obstruction cured by injection of gas, M. Aribund. 207
Iliac, the common, compression of, Dr. Davy................ 208
Impotence and Spermatorrhœa caused by tape worm, M. Houze..................... 204
Instruments new, description of. 658
Insane persons, the law of Illinois concerning, Dr. R. J. Patterson .................. 523
Iodine, solution of, in oil of bitter almonds.............. 556

J.

Jaborandi, therapeutics of .... 106
Jenks, Edw. W., M.D., description of a flexible metallic uterine sound and a flexible metallic probe.............. 661
Jewell, J. S., M.D., on Brown-Séquard and his recent lectures....................... 1

K.

Keitz, Gustav, a case of traumatic tetanus............... 589
Kidney, extirpation of right, Dr. Martin...................... 521
Knee joint, chronic inflammation of, two cases, L. A. Sayre, M.D.............432, 433
Knox, J. Suydam, M.D., the epidemic of small-pox in Chicago during 1877........ 482
Knock-knee, operation for, Prof. Von Nussbaum............ 299

L.

Lead-poisoning by tinned ware and other vessels containing lead, Dr. Kedzie............ 268

PAGE.

Leucocytes, migration of, in passive hyperæmia, W. T. Belfield, M.D. ...................... 247
Leucorrhœa, salicylic acid in... 215
Ligature, a new, E. C. Huse, M. D.......................... 171
Linseed and oil as a therapeutic agent in diseases of skin, Dr. S. Sherwell ................ 381
Lithotomy, two cases of, performed by Dr. Andrews...... 259
Lime water and milk compatible with calomel, Dr. Joynes. 555
Lumbar abscess, a necessity of more heroic treatment, Edmund Andrews, M.D........ 357
Lyman, H. M., M.D.:
  Causes of low rate of mortality among foreign residents at Sandwich Islands......... 22
  An inquiry into the difficulties which hinder a complete registration of births...... 252
  On the code of ethics........ 502
  Paper on fermentation ...... 68
  Correspondence .........182, 398
Lymphatic enlargement of right leg and thigh, sequel and fatal termination of a case of, W. H. Day, M.D., London....... 572
Lungs, œdema of, Dr. W. H. Welch,.................... 94
Laparotomy, Prof. Schröder... 518

M.

Mannheimer, M., M.D., treatment of whooping cough.... 221
McArthur, M.D., death of, resolutions of respect........... 334
McMillan, D. I., M.D., a case of large polypus of the uterus.. 18
Medical education in France, T. K. Cruse, M.D............... 273
Metallotherapy, an additional development of external, Dr. Vigouroux................ 332
Metric system in a nut-shell.... 116
Metric system of weights and measures................... 108
Microphone, illustration of its construction, Dr Tilley..... 271
Miller, DeLaskie, M. D., on treatment of bowel effections of infancy and childhood incident to summer months.... 160
Molluscum contagiosum, three cases of, Roswell Park, M.D. 593
Morphology of the blood in syphilis, lecture by Dr. E. Cutter.. 67

PAGE.

Morphia myosis, explanation of, Dr. Picard.................. 217
Morphia, hypodermic use of, H. L. Harrington, M.D.......... 73
Mortality in the Sandwich Islands, H. M. Lyman, M.D.... 22
Monthly announcements ......112, 224, 336, 448, 560, 672
Myositis ossificans, Dr. Nicoladoni...................... 552

N.

Nasal and uterine hæhmorrage, a new mode of arresting, E. C. Huse, M.D............... 170
Nasal douche, under what conditions it can be employed without danger, translated from Geman of Dr. Leil by F. C. Hotz, M.D............ 280
Necrosis and Osteo-myelitis, drainage of the bones in, M. Armand Després............ 211
Nerve, re-uniting a severed sciatic, M. Wheelhouse..... 551
Neuralgia, epileptiform facial, ammonio-sulphate of copper in..................... 657
Nursing of children, Dr. Brochin 201

O.

Obituary Record .. .......... 111
Obituary of Dr. L. Clark....... 670
Ocular muscles, orthopedic treatment of paralysis of........ 100
Œdema of lungs, Dr. W. H. Welch ...................... 94
Ophthalmology as related to general medicine, E. L. Holmes, M.D............... 143
Os internal, spasmodic contraction of with unusual distension of the cavity of the cervix uteri, M. Bridge, M.D... 487
Ossificans, myositis, Dr. Nicoladoni ...................... 552
Osteoklast, Rizzoli's, Dr. N. Senn ........................ 394
Osteo-myelitis and necroses, drainage of the bones in, M. Armand Després..... ...... 211
Osteo-myelitis during growth, M. Lannelongue............ 211
Otorrhœa, chronic, caries of petrous bone, fatal meningitis, F. C. Hotz, M. D........... 392
Ovarian tumors, malignant and malignant peritonitis, differential diagnosis, Dr. Foulis.. 331

PAGE

Ovary, dermoid tumors of, W. H. Byford, M.D............. 596

P.

Paine, H. M., M.D., the critical period of Homœopathy...... 636
Pancreas, physiology of....... 200
Parametritis, Prof. Breisky's treatment of................. 609
Park, Roswell, M.D.:
Three cases of molluscum contagiosum................. 590
A conspectus of the different forms of consumption .... 228
Parkes, C. T., M.D., unusual high temperature in case of sun stroke...................... 391
Paralysis of ocular muscles, orthopedic treatment of.... ... 100
Paralysis, lesions of the anterior roots in diphtheritic, Déjerine 95
Patterson, R. J., M.D., the law of Illinois concerning insane persons ... ................. 523
Pathology of bone, especially tubercle, Dr. H. H. Smith.... 34
Pathology, the relation of to the motor centers, Dr. W. Kempster....... ............... 64
Patella, fracture of, plaster of Paris dressing for, M. Droullon.......................... 441
Penis, amputation of, Dr. Stocks 209
Peritonitis malignant and malignant ovarian tumors, differential diagnosis, Dr. Foules.... 331
Phthisis, especially its climatic treatment, Dr. A. L. Loomis. 45
Phthisis, tapping a vomica in, Dr. Williams............... 326
Phthisis, contagiousness of, Tappenier...................... 88
Phthisis, antiseptic inhalation in, Eade.................... 550
Phimosis as a cause of hernia.. 400
Pilocarpine, action of eserine and, in hernia of cornea, Dr. F. C. Hotz................. 564
Pityriasis capitis and, of alopecia, treatment of, Malassez... 102
Pityriasis pilaris, M. Richard.. 104
Pneumonia in children, Dr. Monti.................... .. 175
Pneumonia, treatment of in Vienna, W. S. Caldwell, M.D... 180
Polypus of the uterus, case of, D. I. McMillan, M.D. ... 18
Potassium salts, action of, Dr. Buchheim.................. 220

PAGE.
Pregnancy, injection of morphine in vomiting of, M. Chabalier .......... 213
Pregnant woman, obliteration of the uterine neck in a, M. Piron .......... 552
Propylamine in chorea minor, Dr. Pürckhauer .......... 557
Pruritus vulvæ, the relation between diabetes and, Dr. Wiltshire .......... 207
Puerperal fever, the prevention of 90
Puerperal women, the causes of sudden death of, Dr. E. W. Jenks .......... 36
Purpura fatal, following use of iodide of potassium, Mackenzie .......... 430
Pylorus, codeia in the cancer of 657

Q.

Quacks, medical, in Bavaria... 547
Quinia exanthema, Kœbner. ... 106
Quinine, concerning the administration of .......... 656

R.

Rankin, A C., M. D. A case of puerperal convulsions attended with rupture of uterus and recovery .......... 581
Rectum, foreign body in, successful removal .......... 646
Registration of births, an inquiry into the difficulties which hinder a complete, H. M. Lyman, M. D .......... 252
Renal calculus largely composed of indigo .......... 650
Resection of the wrist, M. Reverdin .......... 329
Retina, drainage for detached, Dr. Cohn .......... 216
Retinal, photo-chemistry .......... 648
Reviews and book notices:
Anatomy, descriptive and surgical, Henry Gray, F. R. S. 543
Atlantic islands as resorts of health and pleasure, S. G. W. Benjamin, Esq. .......... 313
Defects of vision, R. B. Carter, F. R. C. S .......... 192
Identification of the human skeleton, Thos. Dwight, M.D .......... 428
Experimental and comparative study of arsenic and cod-liver oil in phthisis pulmonalis, J. Rendu, M.D... 309

PAGE.
Fownes' manual of chemistry. 317
Guide to the practical examination of urine, by James Tyson, M.D .......... 626
Hand-book of ophthalmology, Prof. C. Schweigger.. 537
House drainage and water service in cities etc, I. C. Bayles, Esq .......... 190
How to be plump, by T. C. Duncan, M.D .......... 544
Insanity in ancient and modern life, D. H. Tuke... ... 621
Landmarks, medical and surgical, by Luther Holden, M.D. .......... 428
Law of population, its consequences and bearing upon human conduct and morals, by Annie Besant .......... 619
Lectures on clinical medicine, Dr. M'Call Anderson ...... 408
Lessons in laryngoscopy, including rhinoscopy, by Prosser James, M.D .......... 418
Manual of nursing .......... 422
Nervous diseases, their description and treatment, by Allan McLane Hamilton, M.D .......... 624
Ninth annual report of the State board of health of Massachusetts .......... 419
Personal appearance and the culture of beauty, T. S. Sozinsky .......... 313
Physics of infectious diseases, Dr. C. A. Logan ... ...... 81
Physician's visiting list for 1879, Lindsay & Blakiston. 543
Practical treatise on aural surgery, by H. MacN. Jones.. 310
Practical manual of the diseases of children, with a formulary, Dr. E. Ellis, M.D.. 194
Prescription writing, by F. H. Gerrish, M.D .......... 422
Practical chemistry for medical students, by M. M. P. Muir, F.R.S .......... 195
Practical treatise on diseases of the ear, D. B. St. John Roosa .......... 631
St. Bartholomew's hospital reports, 1877 .......... 425
Synopsis of the diseases of larynx, lungs and heart, F, D. Hall M.D. .......... 193
Transactions of State medical societies:

PAGE.

Transactions of medical and chirurgical faculty of State of Maryland; transactions of the Iowa State medical society; transactions of the 25th annual meeting of medical society of State of North Carolina; transactions of medical association of Alabama; transactions of the medical association of Georgia; transactions of the American gynæcological society for 1877..........315, 540, 627

Urological dictionary, John King, M.D................ 315

Visions, a study of false sight, E. H. Clarke, M. D.........423

Walsh's Physician's Handy ledger, Ralph Walsh, M.D. 632

Rheumatism acute, the use of salicylic acid in, S. W. Gillespie, M.D.................... 148

Richey, S. O., M.D., a modification of the style ............ 356

Rohé, Geo. H., M.D., two cases of syphilis in which infection took place in rather unusual situations .................. 15

Rokitanzy, Karl, Obituary notice of ..................... 223

Rupture of uterus and recovery, attended with puerperal convulsions, A. C. Rankin, M.D. 581

S.

Sacro-iliac disease, differential diagnosis from hip joint disease ....................... 436

Salicylic acid in acute rheumatism, S. W. Gillespie, M.D... 148

Sarcomatous tumor, extirpation of, Prof. Nussbaum......... 297

Schneck, J., M.D., is the bite of the Heterodon or spreading adder venomous............ 585

Scilla, action of, Dr. Huseman.. 219

Sciatica, the nature of, M. Fernet........................ 644

Sclerosis of syphilis, on excision of primitive, Prof. H. Auspitz ..................... 651

Sea-sickness, collodion in, Dr. Laederich.. ............... 325

Sea-sickness, Laederich........ 200

PAGE.

Seguin, Dr., intervention of physicians in education......... 32

Semi-circular canals, the functions of, by H. Gradle, M.D.. 120

Senn, N., Correspondence, surgery in Munich..........297, 394

Septicæmia following resection of bones, Dr. Weeks......... 31

Sciatic nerve, re-uniting a severed, M. Wheelhouse........ 551

Small-pox in Chicago during 1877, J. Suydam Knox, M.D. 482

Society reports:

Chicago Medical Society.... 271

Transactions of Chicago Gynæcological Society........ 596

National Society of Microscopists, reported by W. T. Belfield, M.D............. 265

Michigan State Board of Health, 1878.............. 267

American Medical Association 26

Second annual convention of American Dermatological Association................ 367

Sound, uterine, description of a flexible metallic, by Edw. A. Jenks, M.D................ 661

Spermatorrhœa and impotence caused by tapeworm, Dr. Houze ....... .............. 204

Spondylitis of cervical Vertebræ, L. A. Sayre, M.D....... 431

Scrofuloderm, case of ulcerative, Dr. A. VanHarlingen........ 383

Stewart, J. T., M.D., and Noble Holton, M.D. A case of symmetrical gangrene of the fingers ...................... 587

Stomach, on some forms of chronic catarrh of, Wm. B. Neftel...................... 549

Stomach, chronic catarrh of, Dr. W. B. Neftel................ 206

Style, a modification of the, S. O. Richey, M.D.............. 356

Sunstroke, unusual high temperature in, C. T. Parkes, M.D ...................... 391

Surgical cases, clinical lecture, L. A. Sayre, M.D............. 431

Swan, C. F., M.D., some observations on fifty cases of whooping cough.... ... ......... 154

Syphilis, two cases of, in which infection took place in rather unusual situations, by Geo. H. Rohé, M.D.............. 15

Syphilis, morphology of blood in, E. Cutter, M.D........... 67

PAGE.
Syphilis, on excision of the primitive sclerosis of, Prof. H. Auspitz ................ 651
Syphilitic child, the nurse-maid and the mother of the, J. N. Hyde. M.D ................ 452
Syphiloderm, the pigmentary, I. Edmonson Atkinson, M.D. 340

T.

Talipes, equino-varus, with luxation of knee, L. A. Sayre, M.D .................... 434
Tayuya, physiological action of .................... 555
Tetanus, a case of traumatic, Gustav Keitz, M. D ........ 589
Therapeutics, the bearings of hygiene on, Dr. Black ....... 60
Thomas, H., M.D., an open letter to the *British Medical Journal* ...................... 187
Thrombosis venous, surgical, M. Azam .................... 327
Thyroid gland, extirpation of, Prof. Von Nussbaum ........ 301
Thyroid gland, extirpation of the, Dr. J. T. Miner ........ 38
Tibia, necrosis, two cases, L. A. Sayre, M. D ................ 435
Transplantation of the urethra to the perineum, Prof. Theiroch ...................... 98
Tracheotomy without tubes, Dr. H. H. Martin ............... 31
Tracheotomy, three cases of and recovery in each, D. A. Walden, M.D .................. 76
Tonsurans, the botanical relations of, Dr. I. E. Atkinson .. 381
Tucker, James S, M.D., a visceral æsthesio-neurosis ....... 261
Tuberculosis, etiology of ....... 322
Tumors, dermoid of ovary, W. H. Byford, M.D ............ 596
Typhoid fever of children, Dr. Monti ..................... 176
Typhoid fever, the etiology of, Prof. Huguenin ............ 643

U.

Urea, action of in the blood, Drs. Feltz and Ritter ........ 203
Urethra, female, rapid dilatation of, in the diagnosis and treatment of chronic cystitis, Wm. H. Byford. M.D ........ 10
Uteri, unusual distension of the cavity of the cervix. With spasmodic contraction of the internal os, in a case of miscarriage, N. Bridge, M.D .... 487
Uterine neck, obliteration of in a pregnant woman .......... 552
Uterus:
The influence of in eye disease, Dr. Swanzy .......... 214
Rupture of, attended with puerperal convulsions, and recovery, A. C. Rankin, M.D. 581
A case of partial inversion of, H. T. Byford, M.D ..... 388
Case of large polypus of, D. I. McMillan, M.D ........... 18

V.

Vaccination of very young children, advantage of a single puncture of each arm in, Dr. Hughes .................. 551
Vaginismus, Treatment of, Dr. T. Weber .................. 99
Varicose veins, treatment of, Dr. Englisch .................. 100
Varicocele, new method of treating, Dr. Bradley ............ 210
Vaseline as an excipient ....... 107
Vertigo, the physiology of labyrinthine, Dr. Woakes ........ 548
Visual defects, on some novel modes of relief in, H. Gradle, M.D ...................... 363
Volvulus, Prof. Frerich's treatment ...................... 612
Vomiting during anæsthesia, death from ................ 393

W.

Walden, D. A., M.D., three cases of tracheotomy, recovery in each ...................... 76
Warts, chloral hydrate as a cure for ...................... 657
Waxham, F. E., M.D., aneurism of femoral artery, cured by compression ................ 70
Whooping-cough:
Treatment of, Dr. M. Mannheimer .................. 221
Some observations on fifty cases of, C. F. Swan, M.D.. 154
Bryonia and drasera in the treatment of, Dr. Louvel-Lamare .................. 326

Page.

Wrist, complete re-section of, M. Reverdin ................ 328

Woodworth, Jno. M., M.D., yellow-fever circular........... 558

X.

Xeroderma, a case of so-called Dr. L. A. Duhring........... 369

Page.

Xeroderma, contribut'n to study of, Dr. R. W. Taylor........ 383

Z.

Zinc, oxide of, in diarrhœa .... 555

THE

### Chicago Medical Journal

AND

### EXAMINER.

Vol. XXXVII.—DECEMBER, 1878.—No. 6.

### To our Readers and Correspondents.

Beginning with Vol. XXXVII., July, 1878, the Chicago Medical Journal and Examiner prints all records of length and weight in terms of the Metric System, and all records of temperature in degrees of the Centigrade Scale. The Metric System, legalized in the United States and Great Britain, is extensively or exclusively employed by other civilized nations, and has thus become an essential part of the international language of science. It is recommended for adoption to the profession in this country by the American Medical Association and other scientific bodies. To-day no physician can afford to be ignorant of its value, its simplicity and the meaning of its terms.

The subjoined tables and scales, which have been kindly prepared for us by Prof. W. S. Haines, will be continuously reproduced in subsequent numbers of this journal, for the ready reference of our readers and correspondents.

### Metric Measures of Length.

| | | | |
|---|---|---|---|
| Milimeter | 0.001 | of a Meter | 0.03937 inches. |
| Centimeter | 0.01 | " " | 0.39370 " |
| Decimeter | 0.1 | " " | 3.93707 " |
| **Meter** | **1.** | **Meter** | **39.37079** " |
| Decameter | 10. | Meters | 393.70790 " |
| Hectometer | 100. | " | 3937.07900 " |
| Kilometer | 1000. | " | 39370.79000 " |

### Metrical Weights.

| | | | |
|---|---|---|---|
| Milligram | 0.001 | of a Gram | 0.015 grains. |
| Centigram | 0.01 | " " | 0.154 " |
| Decigram | 0.1 | " " | 1.543 " |
| **Gram** | **1.** | **Gram** | **15.432** " |
| Decagram | 10. | Grams | 154.323 " |
| Hectogram | 100. | " | 1543.234 " |
| Kilogram | 1000. | " | 15434.348 " |

☞ The United States "nickel" five cent piece weighs five grams, and is two centimeters in diameter.

### Approximate Equivalent of Metrical Weights.

*For Rapid Reference.*

| *Milligrams.* | *Grains.* | *Decigrams.* | *Grains.* |
|---|---|---|---|
| 1 (written 0.001 or \|001)* | $^1/_{65}$ | 1 (written 0.1 or \|1) | $1^1/_2$ |
| 2 | $^1/_{32}$ | 2 | 3 |
| 3 | $^1/_{22}$ | 3 | $4^1/_2$ |
| 4 | $^1/_{16}$ | 4 | 6 |
| 5 | $^1/_{13}$ | 5 | $7^1/_2$ |
| 6 | $^1/_{11}$ | 6 | 9 |
| 7 | $^1/_9$ | 7 | 11 |
| 8 | $^1/_8$ | 8 | 12 $/_2$ |
| 9 | $^1/_7$ | 9 | 14 |

| *Centigrams.* | *Grains.* | *Grams.* | *Grains.* |
|---|---|---|---|
| 1 (written 0.01 or \|01) | $^1/_6$ | 1 (written 1. or 1\|) | 15 |
| 2 | $^1/_3$ | 2 | 30 |
| 3 | $^6/_{13}$ | 3 | 46 |
| 4 | $^7/_{11}$ | 4 | 61 |
| 5 | $^3/_4$ | 5 | 77 |
| 6 | $^9/_{10}$ | 6 | 92 |
| 7 | 1 | 7 | 108 |
| 8 | $1^1/_4$ | 8 | 123 |
| 9 | $1^1/_3$ | 9 | 139 |

A Kilogram=$2^1/_5$ lbs. Avoirdupois.

* The decimal *line* instead of points makes errors impossible.

### METRIC FLUID MEASURES.

When using the metric system, fluids are preferably prescribed by weight, employing the gram, its multiples and subdivisions, just the same as with solids, thus avoiding the errors due to refraction, adhesion, and inaccurate measuring vessels. For practical purposes four grams of water may be regarded as equivalent to a fluid drachm of that liquid, and the same may be considered true of tinctures and infusions; syrups, on the average, are about one-third heavier than water, so that a fluid ounce of a syrup will be approximately represented by 43 grams.

If preferred, however, fluids may be prescribed by volume in the metric, just as in the present system, using for that purpose the *Cubic Centimeter*, that is, a volume represented by a cube all of whose sides measure one centimeter. An ordinary back-gammon die is usually about this size. One cubic centimeter (written 1 C. C.) = 16.231 minims. It is approximately regarded as one fourth of a fluid drachm.

### APPROXIMATE EQUIVALENTS OF CUBIC CENTIMETER.

| | | | | |
|---|---|---|---|---|
| 0.001 | C. C. | = ......... | $^1/_{65}$ | minim. |
| 0.01 | " | = ......... | $^1/_6$ | " |
| 0.1 | " | = ......... | $1^1/_2$ | " |
| **1.** | **C. C.** | **= ..................** | **15** | **minim.** |
| 4. | " | = ......... | 1 | fluid drachm. |
| 16. | " | = ......... | 4 | fluid drachms. |
| 32. | " | = ......... | 1 | ounce. |

1000 C. C. (usually known as a **Liter**) is a trifle more than one quart, wine measure.

The following prescription—

R: Potassii bromidi, ℥i.
Elixir aurantii, fl., ℥viij.
M.

Would, in metric terms, be written:

Potassic bromide, 32

Orange elixir, 250

### TO OUR SUBSCRIBERS.

As the present subscription of many of our readers will terminate with this number, we wish to call their attention to the fact that in order to make the journal most effective for benefiting the profession, it is absolutely necessary to keep it on a sound financial basis, and the experience of medical journals the world over shows that it is impossible to do this without making subscriptions payable in advance. We, therefore, urge our patrons to send in their renewals before our next mailing day, in order to insure the uninterrupted receipt of their journals. We always receipt promptly; therefore, if any one does not hear from his remittance within ten days, he may fairly suppose it has gone astray, and he should write us immediately, giving date of remittance, etc.

We have made arrangements which, we think, will be for the advantage of those of our subscribers who wish their journals bound, or who may desire to purchase medical books. For particulars, please read our notice among the advertising sheets.

It greatly facilitates the work of book-keeping for the physician. It is arranged with lines and columns on the one page for the number of calls, and corresponding lines and columns on the opposite page for the necessary particulars. Those who meditate a change in their system of booking will do well to become acquainted with it. R. T.

---

### BOOKS AND PAMPHLETS RECEIVED.

Record of Medical Observations. XXIII edition. (Prescription blanks; metric system.) New York. Edward Seguin, M.D.

Notes on the Treatment of Skin Diseases. By Robert Liveing, A.M., M.D. 4th edition. Revised and Enlarged. 12mo., cloth; pp. 127. New York: W. Wood & Co. 1878

Diseases of the Bladder and Urethra in Women. By A. J. C. Skene, M.D. Cloth; 8vo., pp. 379. New York: W. Wood & Co. 1878.

Walsh's Physicians' Handy Ledger: a Compilation of Walsh's Physicians' Combined Call Book and Tablet. By R. Walsh, M.D., Washington, D. C. Chicago: W. T. Keener, 94 Washington street.

Walsh's Physicians' Combined Call Book and Tablet. 4th edition. W. T. Keener, 94 Washington street, Chicago.

The Illinois State Medical Register for 1878–9. Published annually, under the supervision of the Chicago Medico-Historical Society, with the co-operation of the Ill. State Med. Society. By D W. Graham, M.D. Vol. IV. Chicago: W. T. Keener, 94 Washington street.

Medical and Surgical Directory of the State of Iowa for 1878–9. By Ch. H. Lothrop, M. D. Cloth; pp. 155.

Fifth Annual Report of the State Board of Health of the State of Michigan for the Fiscal Year ending Sept. 30, 1877. Cloth; pp. 503.

Physician's Pocket Day Book. By C. H. Leonard, M. D. Chicago: Jansen, McClurg & Co.

A Therapeutical Inquiry into Rational Medicine. By S. W. Whitmore, M.D.

Case of Sarcoma of the Kidney in a Negro Child. By W. H. Gedding, M. D. Reprint from Gynæcological Transactions.

State of Illinois, Cook County, Circuit Court; Decision rendered by Hon. E. S. Williams at the October term of Court, 1878; Nathan J. Aiken vs. State Board of Health. Twenty copies.

Report on Malpractice. A paper read before the Maine Medical Association June 12, 1878. By Eugene F. Sawyer, A. M., M. D.

Clinical Lectures on Surgery, delivered at Starling Medical College. By J. H. Pooley, M. D. Reprint from *Ohio Medical and Surgical Journal.*

Forms for the Taking of Throat Cases, with Outlines of Fauces and Posterior Nares and of Larynx, as seen in the Mirror, and with a Tabular Statement of the Differential Signs of Various Laryngeal Diseases. Also, for the Taking of Aural Cases, with Drawing and Outlines of the Membrana Tympani, of Fauces and of Posterior Nares, and with a Short Explanation of the Significance of Various Aural Symptoms. By Lennox Browne, F. R. C. S., editor.

Treatment of the Fracture of the Shaft of the Femur. The American Method. A paper read before the Minnesota State Medical Society, at St. Peter, June 18, 1878. By Franklin Staples, M. D. Reprint from the Transactions of the Society, 1878.

The Spontaneous and Artificial Destruction and Expulsion of Fibrous Tumors of the Uterus. By W. H. Byford, M. D. Reprint from Vol. I, Gynæcological Transactions, 1876.

Fibrous Tumors of the Uterus. By W. H. Byford, M. D., Chicago. Vol. III, No. VII of a Series of American Clinical Lectures. Edited by Ed. Seguin, M. D.

Medical Missions at Home and Abroad. By J. G. Kerr, M. D.

Prevention of Disease, Insanity, Crime and Pauperism. A paper read before the Conference of Charities at Cincinnati, May 27, 1878. By Nathan Allen, M. D.

Observations on Nasal Catarrh and Catarrhal Deafness. By W. A. Williamson, M. D.

Ca e of Poisoning by Oil of Chenopodium. By Thomas R. Brown, M. D. Reprint from the *Maryland Medical Journal* for Nov., 1878.

The Relation of Ozone to Disease: Prize Thesis. By J. F. Baldwin, M. D. Reprint from *Ohio Medical and Surgical Recorder*, Oct., 1878.

Discours Prononcé à la re-ouverture des Cours, le 1st Octobre, 1878. Par T. E. D'Odet D'Orsonneus, M. D.

Restoration of the Membrana Tympani. By S. O. Richey, M. D. Extracted from the Transactions of the Illinois State Medical Society. Springfield, May, 1878.

The Nurse Maid and Mother of the Syphilitic Child. (A Clinical Lecture delivered at the Dermatological and Venereal Clinic, Rush Medical College.) By James Nevins Hyde. Reported by L. C. Waters, clinical assistant.

Restriction and Prevention of Diphtheria. Document issued by the State Board of Health of Michigan.

Relative Frequency of Color Blindness in Males and Females. By B. Joy Jeffries, A. M., M. D. Reprint from the *Boston Medical and Surgical Journal*, July 25, 1878.

The Local Treatment of Eczema. By Henry G. Piffard, M. D. Reprint from *The Medical Record*, Oct. 26, 1878.

On Gastro-Elytrotomy. By Henry J. Garrigues, M. D. With three woodcuts. Reprint from *The New York Medical Journal*, Oct. & Nov., 1878.

Apparatus for Transfusion. Asphyxia in New-Born Children Considered from a Medical and Legal Standpoint. By H. J. Garrigues. Reprint from *American Journal of Obstetrics and Diseases of Women and Children*, Vol. XI, No. IV, Oct., 1878.

---

Too Liberal Indeed ! —We read in the *Medical Press and Circular*, of an extraordinary liberality on the part of London hospitals, which we would not advise our hospital authorities to adopt: " The out-patient department of St. Thomas' Hospital is very extensive, the total attendances during the last year being nearly 140,000. Some of these patients come from considerable distances, and are necessarily detained at the hospital, waiting their turn in rotation for medicine, etc. It frequently happens that they become faint before leaving the hospital. In view of this, the treasurer and committee have made arrangements for supplying them with refreshments, such as tea, coffee, milk, rolls, cakes, etc., at a small fixed charge, so that such as require it may obtain a single meal at a small cost. * * * Somewhat similar arrangements have been adopted at Guy's Hospital."

As the *Press and Circular* says : " There is such a thing as being too kind and we should like to be sure that such hospitality will not rather attract than keep away those applicants who can well afford to pay for the advice they require."

A fact of some medico-legal importance is asserted by Dr. Géllè. The tympanic cavity of a fœtus at term is filled with a gelatinous fluid. If a child has breathed an hour or two, the tympanum will be found to contain air.—*Med. Press & Circular.*

### ANNOUNCEMENTS FOR THE MONTH.

### SOCIETY MEETINGS.

Chicago Medical Society—Mondays, Dec. 9 and 23.
West Chicago Medical Society—Mondays, Dec. 16 and 30.

### CLINICS.

MONDAY.

Eye and Ear Infirmary—2 p. m., Ophthalmological, by Prof. Holmes; 3 p. m., Otological, by Prof. Jones.
Mercy Hospital—1:30 p. m., Surgical, by Prof. Andrews.
Rush Medical College—2 p. m., Dermatological and Venereal, by Dr. Hyde; 3 p. m., Medical, by Dr. Bridge.
Woman's Medical College—2 p. m., Dermatological, by Dr. Maynard.

TUESDAY.

Mercy Hospital—1:30 p. m., Medical, by Prof. Hollister.

WEDNESDAY.

Chicago Medical College—1:30 p. m., Eye and Ear, by Prof. Jones.
Eye and Ear Infirmary—2 p. m., Ophthalmological, by Dr. Hotz.

THURSDAY.

Chicago Medical College—1:30 p. m., Medical, by Prof. Quine.
Rush Medical College—3 p. m., Diseases of the Nervous System, by Prof. Lyman; 4 p. m., Diseases of the Chest, by Prof. Ross.

FRIDAY.

Mercy Hospital—1:30 p. m., Medical, by Prof. Davis.

SATURDAY.

Rush Medical College—2 p. m., Surgical, by Prof. Gunn.
Chicago Medical College—2 p. m., Surgical, by Prof. Isham; 3 p. m., Neurological, by Prof. Jewell.
Woman's Medical College—11 a. m., Ophthalmological, by Dr. Montgomery.

Daily Clinics, from 2 to 4 p. m., at the Central Free Dispensary, and at the South Side Dispensary.

